# The largest and heaviest giant juvenile fibroadenoma of the breast in the Chinese population: A case report

**DOI:** 10.1097/MD.0000000000033422

**Published:** 2023-03-31

**Authors:** Yuanxin Zhang, Junhua Huang, Ling Zhou, Ying Leng

**Affiliations:** a Geriatric Diseases Institute of Chengdu/Cancer Prevention and Treatment Institute of Chengdu, Department of Breast Surgery, Chengdu Fifth People’s Hospital (The Second Clinical Medical College, Affiliated Fifth People’s Hospital of Chengdu University of Traditional Chinese Medicine), Chengdu, China.

**Keywords:** case report, Chinese population, giant juvenile fibroadenoma

## Abstract

Fibroadenoma is the most common benign tumor of the breast, but giant juvenile fibroadenoma exceeding 20 cm is much rare. This report presents the largest and heaviest giant juvenile fibroadenoma in an 18-year-old Chinese girl.

**Diagnosis and interventions:**

An 18-year-old adolescent girl with a 2-year history of a large left breast mass with progressive expansion over 11 months. A 28 × 21 cm soft swelling occupied the entire outer quadrants of the left breast. The huge mass sagged below the belly button, resulting in high asymmetry of the shoulders. Contralateral breast examination results were normal except for hypopigmentary detected on the nipple-areola complex. Under general anesthesia, the lump was completely excised along the outer envelope of the tumor, while reserving excessive resection of the skin. The patient’s postoperative recovery was uneventful, and the surgical wound healed well.

**Outcomes:**

A radial incision operation was finally performed to remove the huge mass and to preserve the normal breast tissue and the nipple-areolar complex, not only considering the aesthetics but also preserving the ability to lactate.

**Lessons:**

Currently, there is a lack of clear guidelines regarding the diagnostic and treatment modalities for a giant juvenile fibroadenoma. The principle of surgical choice is to balance aesthetics and function preservation.

## 1. Introduction

Breast fibroadenoma is the most common benign breast tumor, accounting for 7% to 13% of breast outpatients, and can occur in women of any age after puberty.^[1]^ A juvenile fibroadenoma is considered “giant” if it is >5 cm, 500 g, or replaces at least 80% of the breast.^[2,3]^ There are reports of juvenile fibroadenomas in female patients from 9 to 25 years of age^[2]^, This type accounts for 1% to 8% of breast tumors in adolescent women, and the tumors grow rapidly, which can cause bilateral breast asymmetry, breast discomfort, and increase the psychological burden of patients. Its rapidly increasing biological behavioral characteristics also can cause clinical misdiagnosis in patients.^[4]^ For most patients are in adolescence, the treatment method needs to take the aesthetics and the function of breast feeding into account. That is, to preserve normal glandular tissue to the greatest extent and avoid damage to the nipple and areola.^[5]^ Here we describe a largest and heaviest giant juvenile fibroadenoma in an 18-year-old Chinese girl and we want to highlight the minimal surgical trauma. This manuscript is written following CARE checklist.

## 2. Case description

An 18-year-old adolescent girl presented to the clinic with a 2-year history of a large left breast mass with progressive expansion over 11 months. She denied any pain, breast discharge, or systemic signs or symptoms. She had no known family history of breast or ovarian cancer. Menarche was at 13 years and regular. The patient denied any sexual activity.

**Figure 1.**
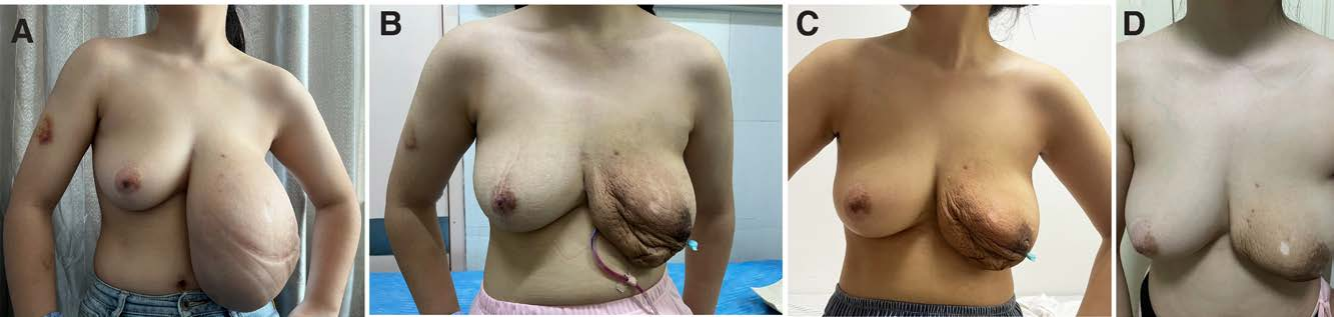
Preoperative and postoperative view of the bilateral breasts. A huge mass on the left breast sagging below the belly button. Hypopigmentary was detected on the nipple-areola complex (A). Postoperative view 5 d after surgery (B). Nine d after surgery. (C). 40 d after surgery (D).

On physical examination, she was found to have obvious asymmetry of the breasts, with the left being significantly larger. Referring to the Regnault grading method, breast sagging was “severe.” There was a 28 × 21 cm soft swelling on the left breast occupying the entire outer quadrants of the left breast. The huge mass sagged below the belly button, resulting in high asymmetry of the shoulders. Contralateral breast examination results were normal except for hypopigmentary detected on the nipple-areola complex (Fig. 1A).

**Figure 2.**
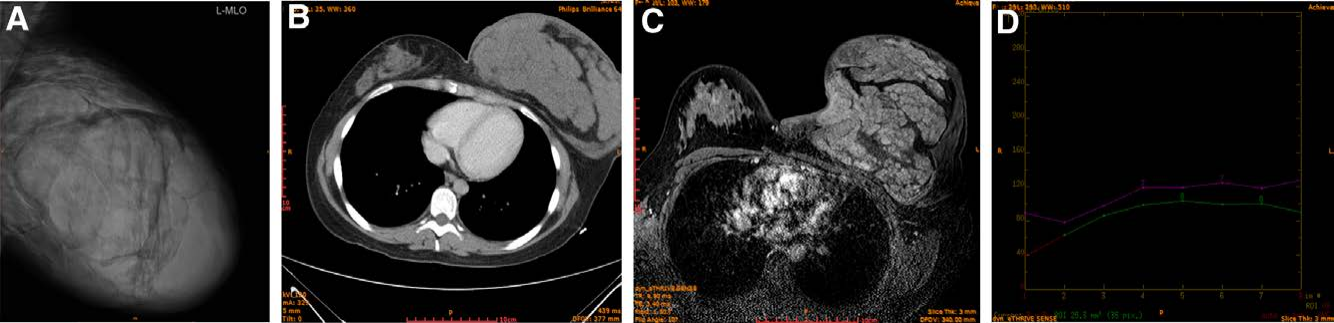
Preoperative imaging data of breasts. Mediolateral oblique mammogram (A). Computed tomography (CT) scan (B). Magnetic resonance imaging (MRI) scan (C).

Her complete blood count revealed anemia (hemoglobin:10 g/dL). An ultrasound scan showed a solid, hypoechoic lesion in the left breast. The mediolateral oblique mammogram showed a 27 cm × 20 cm × 15 cm mass occupying almost the entire left breast (Fig. 2A). A computed tomography scan of the chest and a magnetic resonance imaging scan were also done. Findings from both were suggestive of a large proliferative mass lesion (12.8 cm × 24.1 cm × 18.3 cm) involving the entire breast parenchyma with preserved musculofascial planes, and the time-signal curve is continuously rising (Fig. 2B–D). A core biopsy was performed of the left side that confirmed the lump to be a fibroadenoma. She got a low BREAST-Q score, with symptoms such as sleep disorders, depression, inattention, and so on.

**Figure 3.**
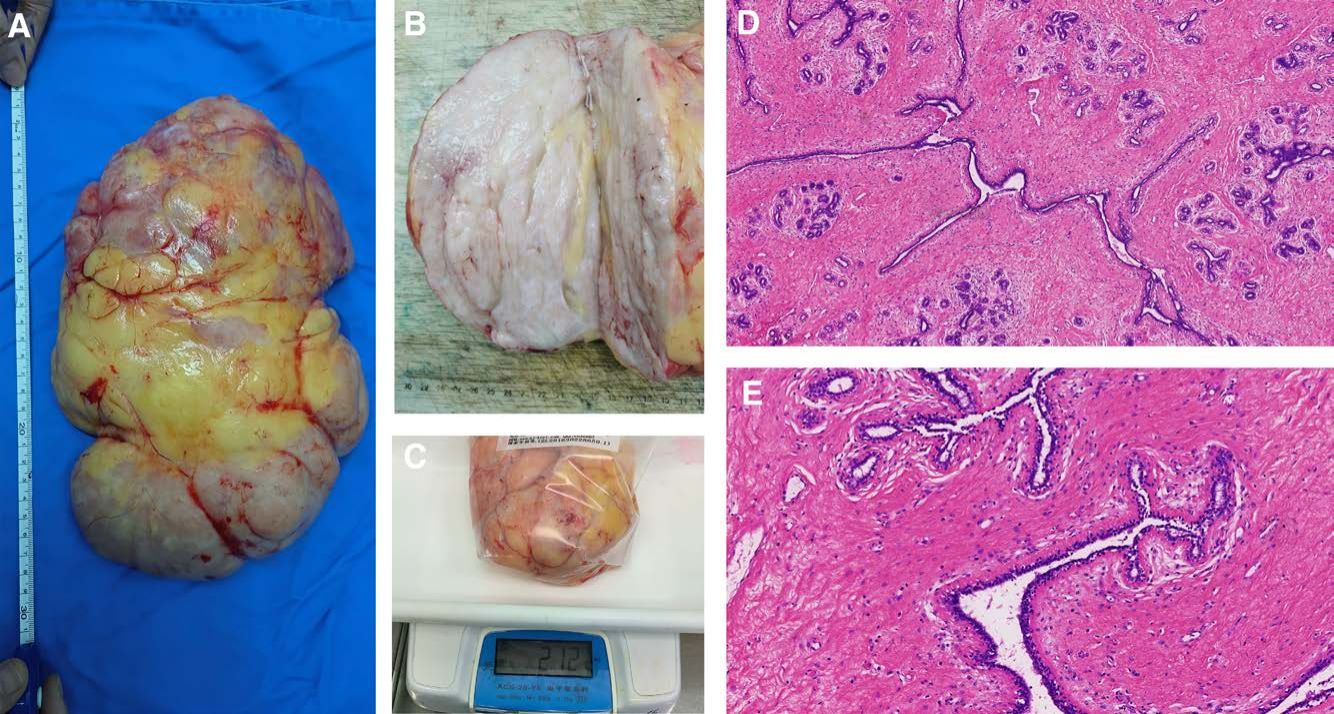
The huge mass removed by surgery. Size of the tumor was 29.4 cm × 21.0 cm × 12.0 cm (A). Transverse section of the tumor (B). Weight of the tumor (C). Incisional biopsy showing leaf-like architecture (40×) (D), with epithelial and stromal overgrowth (100×) (E).

After extensive counseling of the patient and her parents, a decision was made to complete excision or enucleation of the lump. Under general anesthesia, the lump was completely excised along the outer envelope of the tumor, while reserving excessive resection of the skin. The patient postoperative recovery was uneventful, and the surgical wound healed well. The final histopathological report revealed a fibro-epithelial tumor 29.4 cm × 21.0 cm × 12.0 cm, which was well circumscribed and reached all resected margins (Fig. 3A and B). The mass weighed 2.12 kg (Fig. 3C). There was an abundance of the epithelial component along with hypercellular stroma (Fig. 3D and E). A final diagnosis of juvenile fibroadenoma was made, in keeping with the original biopsy. The patient is in regular follow-up without any recurrent lesions in the past 3 months. The surgery resulted in excellent aesthetics and the patient was very satisfied, she got a high BREAST-Q score when reevaluated postoperatively (Fig. 1B–D). Written consent was obtained from the parents for the procedure and to obtain intraoperative and postoperative photographs.

## 3. Discussion

Giant juvenile fibroadenomas usually present during puberty as encapsulated, rapidly growing breast masses.^[4]^ It is generally believed to be related to excessive estrogen levels and increased estrogen receptor sensitivity.^[6]^ Tumors can grow rapidly in a short period, and their diameter may even exceed 20 cm. Breast skin ulcers, pressure necrosis, and prominent skin dilated veins may appear.^[7]^ Due to the rapid growth of tumors, most of us think that there is a possibility of malignancy, but the cancer rate is <0.3%. The updated literature denotes 60 cm as the largest and 2.1 kg as the heaviest.^[1]^ Islam et al^[8]^ reported a giant juvenile fibroadenoma of the breast in a 16-year-old girl with a 28 × 25 cm mass on her left breast in which breast conservation was done. Jategaonkar et al^[9]^ reported a super-giant juvenile fibroadenoma measured a staggering 63 × 47 cm on the right and left 51 × 39 cm. In our case, it was the largest and heaviest giant juvenile fibroadenoma reported in the Chinese population, hence merited presentation at a global forum.

Histopathological features were mainly characterized by abundant stromal cells and hyperplasia of the glandular epithelium, with more peritubular types than intratubular or mixed types.^[10]^ The glands and interstitium are usually evenly distributed, the extracellular matrix is usually composed of collagen, and necrosis and calcification are not easily seen, the ducts or lobules often have exuberant general hyperplasia, which can be grid-like, papillary, micropapillary, or even solid structure, overlapping nuclei, water-like arrangement, and chromatin condensation; interstitial cells are bipolar spindle cells, with or without mild atypia, and mitotic figures are rare.^[11]^ In rare cases, an interstitial overgrowth can be seen, suggesting the concomitant formation of phyllodes tumor structures.^[12]^

The treatment options are heterogeneous and varied, from simple enucleation to mastectomy with or without immediate or delayed breast reconstruction.^[13]^ The type of incision is also varied from a circumareolar or a sub-mammary incision and incision directly over the lump.^[3,14,15]^ Though encountered rarely, it is quite challenging to manage patients with giant juvenile fibroadenoma. For our patient, we used a radial incision to remove the tumor along the outer envelope of the tumor. The mainstay of treatment is complete excision with an emphasis on preserving the developing breast parenchyma and nipple-areolar complex while reserving excessive resection of the skin. As we all know, there is often some gain in skin elasticity by about 6 months postoperative, so patience is the key before 1 embarks on unnecessary reconstructive procedures. Moreover, as these patients are still undergoing puberty, the breast may develop further and we must keep in mind aesthetics and preserve the ability to lactate.

In this report, we present the largest and heaviest giant juvenile fibroadenoma in an 18-year-old Chinese girl whose tumor was removed successfully by enucleation via a radial incision with the preservation of the normal breast tissue and nipple-areolar complex, both aesthetics and function preservation were realized.

## 4. Conclusions

Detailed clinical, radiological evaluation and accurate differential diagnosis may help in the giant juvenile fibroadenomas management. Currently, there is a lack of clear guidelines regarding the diagnostic and treatment modalities for a giant juvenile fibroadenoma. The mainstay of treatment in such cases is complete excision without damage to the breast parenchyma and nipple-areolar complex. Surgeons should consider not only tumor safety but also the aesthetics and functionality of the breast when developing surgical plans.

## Author contributions

**Conceptualization:** Yuanxin Zhang, Ling Zhou.

**Data curation:** Ying Leng.

**Supervision:** Junhua Huang.

**Writing – original draft:** Yuanxin Zhang.

**Writing – review & editing:** Yuanxin Zhang.
